# Relationship between anterior ankle laxity and anterior talar translation during landing using dual fluoroscopic imaging

**DOI:** 10.3389/fbioe.2026.1934605

**Published:** 2026-09-15

**Authors:** Ye Luo, Feng Li, Xinwei Huang, Qiuyang Xiao, Linguang Feng, Longxiang Li, Yuanbiao Luo, Shaobai Wang

**Affiliations:** 1 School of Exercise and Health, Shanghai University of Sport, Shanghai, China; 2 Department of Rehabilitation Therapy, Yangzhi Affiliated Rehabilitation Hospital of Tongji University, Shanghai, China; 3 Department of Orthopaedics, The First Hospital of Putian City, The School of Clinical Medicine, Fujian Medical University, Putian, Fujian, China

**Keywords:** anterior ankle laxity, anterior talar translation, digital ankle arthrometer, dual fluoroscopic imaging, mechanical stability, single-leg landing

## Abstract

**Objective:**

Ankle sprains are among the most common lower extremity injuries, and anterior ankle laxity may influence dynamic stability during high-impact tasks. Although instrumented measurements under standardized loads provide objective assessment of static anterior ankle laxity, the relationship between passive laxity and *in vivo* dynamic talar motion remains unclear. This study aimed to investigate the association between static anterior ankle laxity and anterior talar translation during a single-leg landing task using dual fluoroscopic imaging.

**Methods:**

Fourteen healthy male participants (age 21.6 ± 1.3 years) performed single-leg landing tasks from a 40 cm platform. Static anterior ankle laxity was measured using a standardized anterior drawer loading protocol with displacement quantified at the 90 N load point. Participant-specific 3D tibia and talus models were reconstructed from MRI scans and registered to dual fluoroscopic images to calculate six-degree-of-freedom talar kinematics. Peak anterior talar translation within the first 100 ms after initial contact was extracted. Pearson correlation analysis assessed the relationship between static anterior ankle laxity and peak anterior talar translation.

**Results:**

Static anterior ankle laxity averaged 7.79 ± 1.32 mm, and peak anterior talar translation during landing was 8.93 ± 1.88 mm, showing notable inter-individual variability. A significant positive correlation was observed between static anterior ankle laxity and peak anterior talar translation (r = 0.818, 95% CI 0.51–0.94, p < 0.001). Ground reaction force data confirmed typical double-peak patterns during landing, and anterior–posterior translation was the primary degree of freedom.

**Conclusion:**

Static anterior ankle laxity is strongly associated with anterior talar translation during high-impact landing, suggesting that passive mechanical properties of the ankle are related to dynamic bone motion. Quantified anterior drawer measurements provide an objective assessment of ankle laxity and may provide biomechanical information relevant to dynamic ankle stability.

## 1 Introduction

Ankle sprains are among the most common lower extremity injuries encountered in sports and daily activities (Herzog et al., 2019). During high-impact movements such as landing, cutting, and jumping, the ankle must withstand substantial ground reaction forces over a short time while maintaining the stability of the ankle complex (Ji et al., 2025). The mechanical stability of the ankle during motion primarily depends on bony architecture, ligamentous constraints, and neuromuscular control (Ji et al., 2025). Among these structures, the anterior talofibular ligament (ATFL), as a key component of the lateral ligament complex, plays a crucial role in limiting anterior translation of the talus relative to the tibia, especially when the ankle is in plantarflexion (Bahr et al., 1997). Inter-individual differences in anterior ankle laxity may affect the magnitude of talar anterior translation during high-impact tasks, further influencing dynamic stability during the landing phase (Brown et al., 2015).

The anterior drawer test (ADT) is commonly used in clinical practice to assess anterior ankle stability. By applying an anterior-directed force and observing talar translation, the test evaluates the mechanical stability of the ankle. Current clinical guidelines recommend measuring and recording anterior talar translation in patients with acute, subacute, or chronic lateral ankle sprains (Martin et al., 2021). However, conventional manual ADT is highly subjective. Test outcomes are susceptible to examiner experience, applied force magnitude, and involuntary muscle guarding by the participant. Systematic reviews also indicate that while the ADT remains clinically valuable for assessing mechanical ankle instability, its diagnostic performance, reliability, and interpretation require combination with standardized procedures and quantitative measures (Luan et al., 2025).

To improve the objectivity of ankle laxity assessment, researchers have increasingly used digital ankle arthrometers to quantify anterior ankle displacement under standardized anterior drawer loading. Unlike manual testing, instrumented arthrometry continuously records load–displacement data, providing a precise and reproducible measure of the ankle’s passive mechanical properties (Chen et al., 2022; Gulick, 2024). This approach allows researchers and clinicians to objectively capture inter-individual differences in anterior ankle laxity that may be difficult to detect with conventional examination methods. Previous studies have shown that these measurements can reliably represent the mechanical behavior of the ankle under applied force (Chen et al., 2022).

However, whether static anterior ankle laxity reflects dynamic stability during functional tasks remains unclear. Landing is a common high-impact movement in sports and a frequent scenario for lateral ankle injuries. During initial contact, the ankle typically transitions from plantarflexion toward dorsiflexion while bearing substantial impact and ground reaction forces. Stability in this phase depends on ligamentous constraints, bony congruence, muscular control, and proprioceptive feedback (Yan et al., 2025). Individuals with greater anterior ankle laxity may exhibit larger anterior talar translation during such dynamic tasks, but this hypothesis requires validation through *in vivo* bone kinematic measurements.

Conventional optical motion capture systems rely on skin-mounted markers to estimate ankle motion, yet the complex anatomy of the foot–ankle region and soft tissue artifacts reduce accuracy for tracking actual bone movement (Luo et al., 2024a). Dual fluoroscopic imaging combined with individualized 3D bone model registration allows direct tracking of bone position and orientation during dynamic tasks, enabling six-degree-of-freedom kinematic analysis of the tibiotalar and subtalar joints (Luo et al., 2025). Roach et al. applied high-speed dual fluoroscopy to study *in vivo* tibiotalar and subtalar kinematics in chronic ankle instability patients and healthy controls during dynamic activities, demonstrating that this approach can reveal bone motion differences that are difficult to detect with conventional motion capture (Roach et al., 2017). Therefore, dual fluoroscopic imaging provides a feasible method to link static laxity measurements with dynamic bone stability.

Based on this background, the present study aimed to combine static anterior ankle laxity measured with a digital ankle arthrometer with anterior talar translation obtained during a single-leg landing task using dual fluoroscopic imaging. We hypothesized that individuals with greater static anterior ankle laxity would exhibit larger anterior talar translation during the landing phase. This study may clarify the relationship between static mechanical laxity and dynamic bone motion of the ankle and provide *in vivo* biomechanical evidence relevant to dynamic ankle stability.

## 2 Materials and methods

### 2.1 Study design

This study was a cross-sectional *in vivo* biomechanical investigation aimed at exploring the relationship between static anterior ankle laxity and anterior talar translation during a single-leg landing task. The study was approved by the Ethics Committee of Shanghai University of Sport (Approval No. 102772021RT133) and adhered to the principles of the Declaration of Helsinki. All participants were fully informed of the study purpose, procedures, and potential risks, and provided written informed consent before participation.

### 2.2 Participants

Fourteen healthy male participants were recruited (age 21.6 ± 1.3 years; height 176.9 ± 4.1 cm; weight 69.9 ± 5.5 kg). All participants engaged in regular physical activity, performing moderate-intensity exercise 2–4 times per week, at a minimum of 30 min per session. All participants were right-leg dominant, as determined using the Waterloo Footedness Questionnaire-Revised (WFQ-R) (van Melick et al., 2017).

Inclusion criteria were: age 18–26 years; body mass index 18.5–23.9 kg/m^2^; no history of ankle injury or chronic ankle instability; no structural ankle abnormalities observed in physical examination or MRI; ability to perform the single-leg landing task. Exclusion criteria were: lower extremity musculoskeletal injury within the past 6 months; prior ankle sprain, surgery, or instability; neurological or psychological conditions affecting motor control; MRI contraindications, such as claustrophobia or metallic implants; inability to complete experimental tasks as instructed. The sample size was comparable to those of previous *in vivo* biplanar fluoroscopy studies of ankle and lower-extremity joint kinematics (Roach et al., 2017; Ye et al., 2023).

### 2.3 Measurement of anterior ankle laxity

Static anterior ankle laxity of the right ankle was measured using a Ligs Digital Arthrometer (Innomotion Inc., China) (Figure 1A). The system was automatically zeroed before testing each participant and was periodically calibrated by the manufacturer. The device continuously recorded force and displacement, with a force resolution of 1 N and a displacement resolution of 0.1 mm (Chen et al., 2022). Participants were positioned supine with the right foot secured in the device and the ankle maintained near neutral (Figure 1B). Participants were instructed to fully relax their lower limb muscles to minimize interference from active muscle contraction (Chen et al., 2022). A standardized anterior drawer load was continuously applied to the hindfoot up to a maximum force of 120 N, simulating the clinical anterior drawer test. Anterior ankle laxity was quantified as the anterior ankle displacement at the standardized 90 N load point during the loading process (Chen et al., 2022). Each participant completed three valid trials, and the mean of the three measurements was used to represent the participant’s anterior ankle laxity under standardized loading conditions.

**FIGURE 1:**
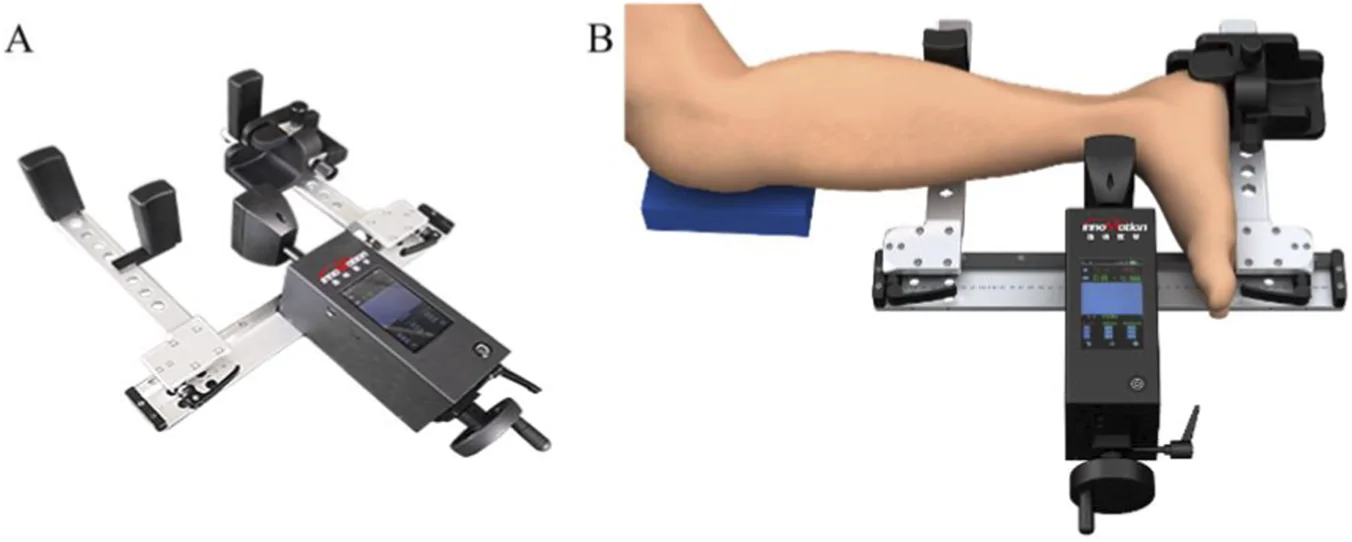
Instrumented measurement of anterior ankle laxity. **(A)** The digital ankle arthrometer used for objective measurement of anterior ankle laxity. **(B)** Participant positioning during the anterior drawer test.

### 2.4 MRI acquisition and three-dimensional bone model reconstruction

All participants underwent right ankle MRI within 48 h before or after the motion capture session. Imaging was performed on a 3.0 T MRI system (MAGNETOM Prisma, Siemens Healthcare, Germany) with participants supine and the right ankle placed near a naturally relaxed neutral position. A T1-weighted three-dimensional sequence was used with the following parameters: repetition time (TR) 10.5 ms, echo time (TE) 4.92 ms, flip angle 10°, slice thickness 0.6 mm, isotropic resolution to ensure precise bone contour reconstruction (Guo et al., 2023).

MRI images were imported into Amira Software, version 2024.1 (Thermo Fisher Scientific, Waltham, MA, United States), for manual slice-by-slice segmentation of the tibia and talus, followed by reconstruction of participant-specific 3D bone models (Figure 2). MRI-based three-dimensional bone reconstruction has previously demonstrated good geometric accuracy and has been applied to ankle joint modeling using Amira (Rathnayaka et al., 2012; Durastanti et al., 2019). The reconstructed tibia and talus models were subsequently used for 2D-to-3D registration with dual fluoroscopic images (Luo et al., 2023).

**FIGURE 2:**
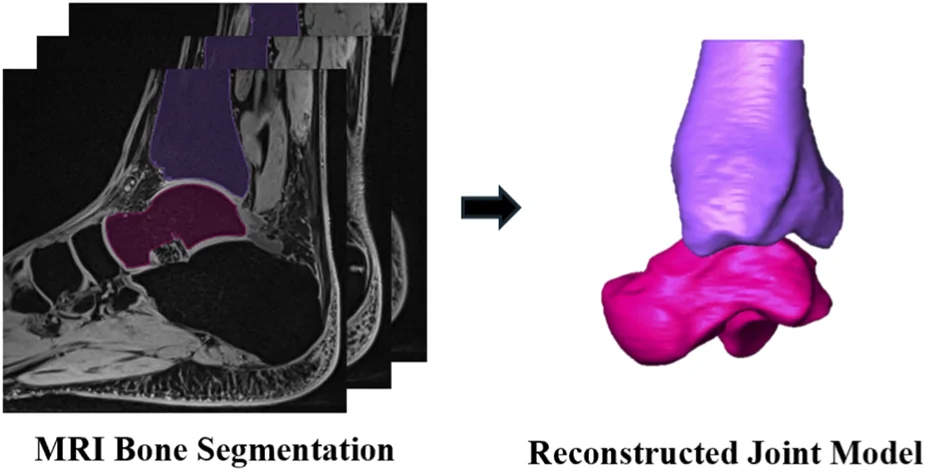
MRI-based bone segmentation and three-dimensional reconstruction.

### 2.5 Single-leg landing task and data acquisition

Single-leg landing experiments were conducted in a biomechanics laboratory. Participants were instructed to avoid strenuous exercise 24 h prior to testing. A standardized warm-up, including 7 min of light jogging, was performed to activate lower limb muscles and reduce injury risk.

Dynamic landing kinematics were recorded using a high-speed dual fluoroscopic imaging system (DFIS), consisting of two X-ray sources and image intensifiers capturing simultaneous images from two angles at 250 frames per second. Previous validation of the DFIS/model-based tracking approach used in this study reported mean translational and rotational errors of 0.03 ± 0.35 mm and 0.25° ± 0.81°, respectively, with corresponding precision values of 0.30 ± 0.12 mm and 0.63° ± 0.28° (Wang et al., 2015). A 3D force plate (Kistler 9286BA, Kistler, Winterthur, Switzerland) sampled ground reaction forces at 1,000 Hz and was synchronized with DFIS via hardware trigger to precisely determine initial contact.

Participants stood on a 40 cm platform and performed a forward step landing with the non-dominant leg (left) leaving the platform first, followed by a stable landing on the right dominant foot (Hanzlíková et al., 2021). Participants were instructed to perform the landing naturally. No specific forefoot, midfoot, or rearfoot strike pattern was prescribed, and ankle posture at initial contact was not externally constrained. Hands were crossed on the chest to minimize upper body motion. Practice trials were allowed until participants were familiar with the task and the right ankle remained within the imaging field. Successful trials required: stable single-foot landing on the force plate, foot–ankle region within fluoroscopic view, no imbalance, no extra steps, no abnormal upper limb motion, and complete synchronized data. When more than one valid trial was available, the trial with the clearest fluoroscopic visualization throughout the analyzed period and complete synchronized data was selected. Consistent with previous high-speed DFIS studies, one technically valid trial per participant was used for the final analysis (Torry et al., 2011; Ye et al., 2023).

### 2.6 Dynamic tibiotalar kinematic analysis

Participant-specific 3D tibia and talus models and the corresponding fluoroscopic images were imported into Rhinoceros 7.0 (Robert McNeel and Associates, Seattle, WA, United States) for 2D-to-3D registration. Manual 2D-to-3D registration was performed frame by frame by adjusting the position and orientation of each bone model until its projected contours matched the corresponding bony outlines in both fluoroscopic views (Figure 3). After registration, six-degree-of-freedom kinematics of the talus relative to the tibia were computed (Luo et al., 2024b). In addition, intra-rater repeat-registration was assessed in two randomly selected participants by repeating the registration of the same landing sequence by the same operator.

**FIGURE 3:**
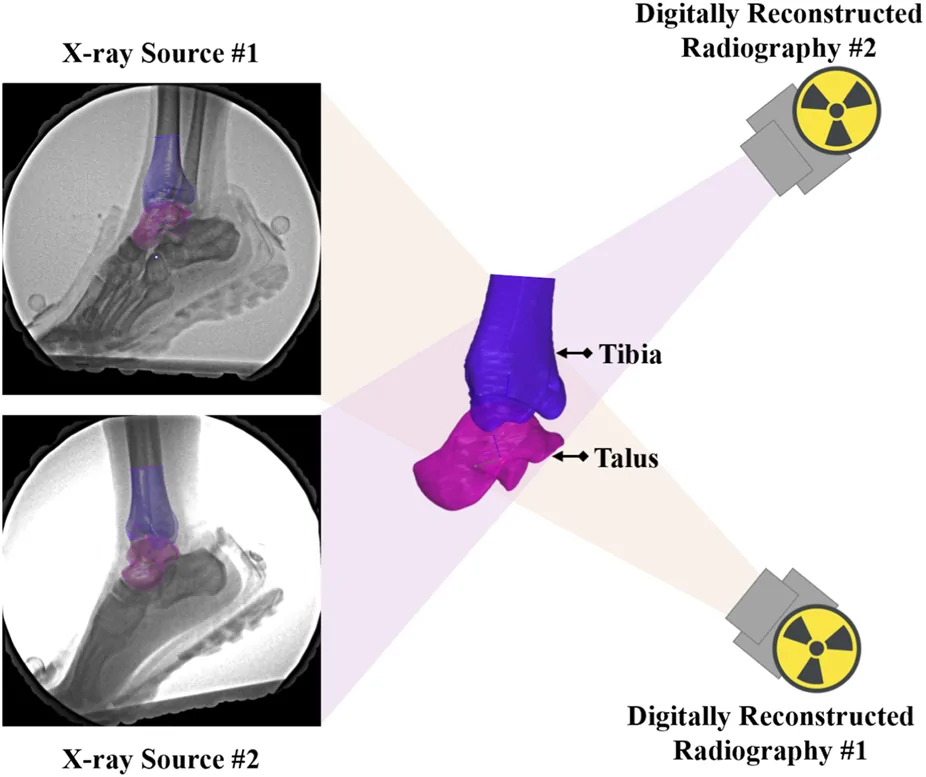
Dual fluoroscopic imaging and 2D-to-3D registration illustration.

Independent coordinate systems were established for each bone following previously reported standards (Yamaguchi et al., 2009; Pitcairn et al., 2020). The X, Y, and Z-axes represent anterior–posterior, medial–lateral, and superior–inferior directions, respectively. Flexion/extension, inversion/eversion, and internal/external rotation were defined about the medial–lateral, anterior–posterior, and superior–inferior axes, respectively. Based on this coordinate system, medial, posterior, and inferior translations were positive; lateral, anterior, and superior translations were negative. Plantarflexion, eversion, and external rotation were positive; dorsiflexion, inversion, and internal rotation were negative. Accordingly, anterior talar translation is represented as negative in anterior–posterior translation curves.

Initial contact was defined as the moment when vertical ground reaction force first exceeded 20 N, designated as 0 ms. The first 100 ms after initial contact was analyzed to characterize the early landing phase, during which rapid joint motion and impact loading occur (Myers et al., 2011). Peak anterior talar translation within this interval was extracted; the absolute magnitude of the minimum value on the anterior–posterior translation curve was defined as peak anterior translation.

### 2.7 Statistical analysis

Statistical analyses were performed using IBM SPSS Statistics (Version 26.0, IBM Corp., Armonk, NY, United States). All continuous variables are presented as mean ± standard deviation (mean ± SD). Normality of static anterior ankle laxity and peak anterior talar translation during the landing task was assessed using the Shapiro–Wilk test (Shapiro and Wilk, 1965). For normally distributed data, Pearson correlation analysis was conducted to evaluate the relationship between static anterior ankle laxity and peak anterior talar translation. The 95% confidence interval (CI) for Pearson’s correlation coefficient was calculated using Fisher’s z transformation. The correlation coefficient r was used to indicate correlation strength: 0.00–0.30 negligible, 0.30–0.50 low, 0.50–0.70 moderate, 0.70–0.90 high, and 0.90–1.00 very high (Mukaka, 2012). Statistical significance was set at α = 0.05 using two-tailed tests. For the repeat-registration assessment, agreement in anterior–posterior talar translation was quantified using the mean absolute difference, root-mean-square difference, and absolute difference in peak anterior talar translation.

## 3 Results

### 3.1 Ground reaction force during single-leg landing

All participants successfully completed the high platform single-leg landing task and were included in the analysis. The vertical ground reaction force (vGRF) exhibited a typical double-peak pattern after landing. The first peak occurred at 11.43 ± 1.65 ms after initial contact, with a magnitude of 1.258 ± 0.324 times body weight. The second peak occurred at 43.43 ± 5.94 ms, with a magnitude of 3.545 ± 0.536 times body weight.

### 3.2 Tibiotalar joint kinematics during landing

During the first 100 m after initial contact, the talus exhibited pronounced translational and rotational movements relative to the tibia. The anterior–posterior direction was the primary translational degree of freedom, with a range of motion (ROM) of 16.65 ± 1.85 mm. Medial–lateral translation ranged 3.13 ± 0.58 mm, and superior–inferior translation ranged 2.95 ± 1.16 mm. Rotationally, plantarflexion–dorsiflexion was dominant with a ROM of 42.53° ± 4.74°, inversion–eversion ROM was 6.15° ± 1.73°, and internal–external rotation ROM was 9.07° ± 3.33° (Table 1). The repeat-registration assessment showed a pooled mean absolute difference of 0.27 mm and a root-mean-square difference of 0.34 mm for anterior–posterior talar translation, with absolute differences in peak anterior talar translation of 0.23 and 0.26 mm in the two participants.

**TABLE 1:** Tibiotalar joint kinematics during the first 100 ms after initial contact.

Motion of the talus relative to the tibia	Maximum	Minimum	ROM
Medial or lateral translation	1.35 ± 0.45 mm	−1.78 ± 0.64 mm	3.13 ± 0.58 mm
Posterior or anterior translation	7.72 ± 2.16 mm	−8.93 ± 1.88 mm	16.65 ± 1.85 mm
Inferior or superior translation	−0.10 ± 0.42 mm	−3.05 ± 0.94 mm	2.95 ± 1.16 mm
Plantarflexion or dorsiflexion	23.07° ± 4.80°	−19.46° ± 5.78°	42.53° ± 4.74°
Eversion or inversion	2.46° ± 1.84°	−3.69° ± 1.71°	6.15° ± 1.73°
External or internal rotation	6.56° ± 2.27°	−2.51° ± 2.44°	9.07° ± 3.33°

Values are presented as mean ± standard deviation. Positive values indicate medial, posterior and inferior translations, as well as plantarflexion, eversion and external rotation. Negative values indicate lateral, anterior and superior translations, as well as dorsiflexion, inversion and internal rotation.

### 3.3 Relationship between anterior ankle laxity and anterior talar translation

At the standardized 90 N load point during anterior drawer loading, static anterior ankle laxity was 7.79 ± 1.32 mm, showing inter-individual variability. During the first 100 ms of the single-leg landing task, peak anterior talar translation relative to the tibia was 8.93 ± 1.88 mm, also exhibiting notable inter-individual variability. Pearson correlation analysis revealed a significant positive correlation between static anterior ankle laxity and peak anterior talar translation during landing, with a correlation coefficient of r = 0.818 (95% CI, 0.51–0.94; p < 0.001) (Figure 4).

**FIGURE 4:**
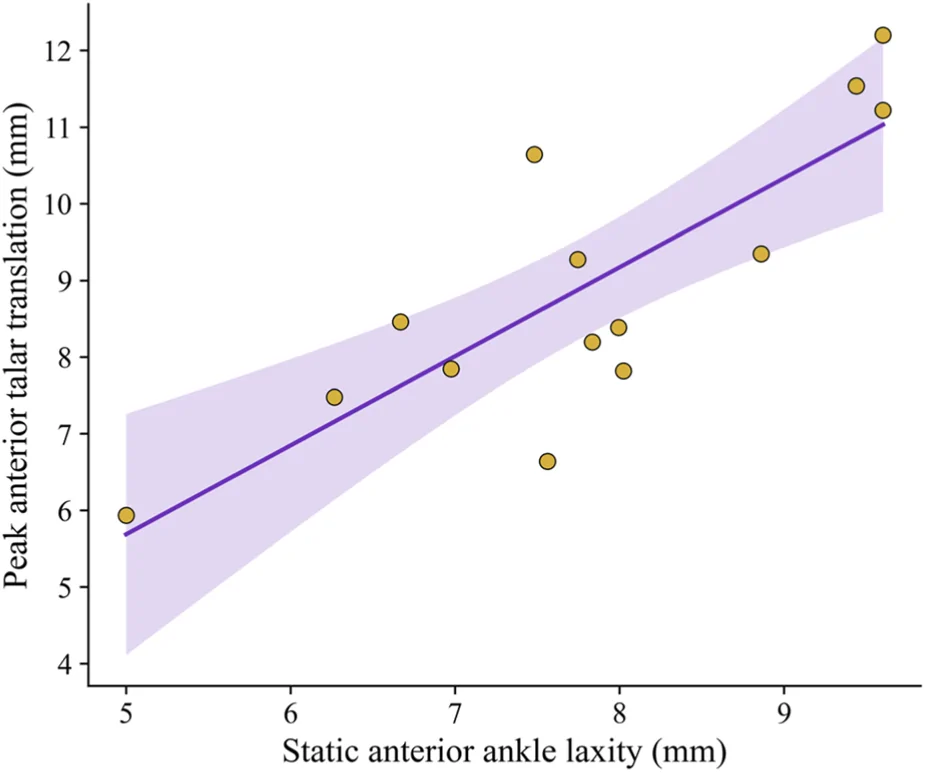
Relationship between static anterior ankle laxity and peak anterior talar translation during landing. Static anterior ankle laxity was quantified as anterior ankle displacement at the standardized 90 N load point during anterior drawer loading, and peak anterior talar translation was quantified during the first 100 ms after initial contact. Each point represents one participant. The solid line represents the linear regression fit, and the shaded area represents the 95% confidence band.

## 4 Discussion

This study systematically evaluated the relationship between static anterior ankle laxity and dynamic anterior talar translation relative to the tibia during a single-leg landing task. This study found a significant positive correlation: individuals exhibiting greater anterior ankle displacement at the standardized 90 N load point also demonstrated larger peak anterior talar translation within the first 0–100 ms after landing. To our knowledge, this is the first study to directly quantify the relationship between static ankle laxity and *in vivo* bone motion during a high-impact task using dual fluoroscopic imaging combined with participant-specific 3D bone model registration, providing important evidence for understanding the association between mechanical ankle stability and dynamic bone kinematics.

Passive mechanical stability of the ankle is primarily maintained by bony congruence, the joint capsule, and the ligamentous complex, among which the anterior talofibular ligament (ATFL) is a key structure restricting anterior talar translation relative to the tibia (Bahr et al., 1997). Previous studies have shown that injury to the lateral ankle ligament complex is associated with increased mechanical laxity and alterations in ankle joint kinematics (Kerkhoffs et al., 2002; Canton et al., 2020). In this study, anterior ankle laxity was quantified as anterior ankle displacement at the standardized 90 N load point using a digital arthrometer, reflecting the passive mechanical displacement characteristics of the ankle. Correlation analysis indicated that this static measure was closely associated with peak anterior talar translation during dynamic landing (r = 0.818, p < 0.001). One possible explanation is that greater passive laxity may permit greater talar motion under high-impact loading conditions. However, the observed correlation corresponded to an r^2^ of 0.669, indicating that approximately one-third of the variance in peak anterior talar translation remained unexplained by static anterior ankle laxity alone. Dynamic talar motion also depends on neuromuscular control and other biomechanical factors; therefore, passive laxity should not be considered the sole determinant of dynamic stability.

Conventional manual ADT is limited by examiner variability, inconsistent applied force, and participant muscle tension, resulting in subjective outcomes (Martin et al., 2021). Systematic reviews also indicate that although the test retains value for assessing mechanical ankle instability, reliability and clinical interpretation should be supported by quantitative measures (Croy et al., 2013). Recently, instrumented arthrometers under standardized loading conditions have been shown to objectively quantify anterior ankle laxity as continuous variables and exhibit high diagnostic efficacy for chronic ankle instability (AUC ≈0.92) (Chen et al., 2022). Gulick also highlighted that instrumented measurement enhances objectivity in ankle laxity assessment (Gulick, 2024). Our findings extend this framework by demonstrating a significant association between quantified anterior ankle laxity and dynamic talar motion during high-impact functional tasks. These findings suggest that quantified anterior ankle laxity may provide biomechanical information relevant to dynamic ankle stability. Whether this measure has value for injury-risk assessment or clinical evaluation requires further investigation in prospective and clinical populations.

Similar relationships have been observed in other joints such as the knee. Torry et al. used dual fluoroscopy to evaluate 16 healthy adults and found a significant positive correlation between passive anterior knee laxity measured by KT-1000 and peak anterior tibial translation during a 40 cm drop-landing task (group r = 0.89, p < 0.0001; females r = 0.93; males r = 0.97) (Torry et al., 2011). The current study reproduced a similar pattern at the ankle joint, with a correlation coefficient (r = 0.818) also within the high correlation range, suggesting that a similar relationship between passive laxity and dynamic bone translation may occur across different joints. Additionally, a prospective study in West Point cadets reported that higher knee anterior laxity and generalized joint laxity were associated with increased risk of non-contact ACL injury, suggesting the potential predictive value of laxity measurements for injury (Uhorchak et al., 2003). Although these findings support the broader relevance of passive joint laxity, the present cross-sectional ankle study does not establish predictive validity for future injury. Prospective studies are needed to determine whether quantified anterior ankle laxity is associated with subsequent functional instability or ankle injury risk.

This study has several limitations. First, the sample size was small (n = 14), and all participants were healthy young males, limiting the generalizability of the findings to females, older individuals, populations with different activity levels, and clinical populations. In particular, patients with chronic ankle instability or those undergoing post-injury recovery were not included; therefore, the strength or pattern of the observed relationship under pathological conditions cannot be directly inferred. Females may also exhibit different ligament laxity and landing strategies, requiring further investigation. Second, the cross-sectional design and absence of prospective injury or clinical outcome data preclude conclusions regarding the predictive validity of anterior ankle laxity for future functional instability or ankle injury. Third, foot-strike pattern and ankle posture at initial contact were not prospectively standardized. Although all participants were observed to contact the ground with the forefoot first and were in plantarflexion at initial contact (22.99° ± 4.73°), inter-individual variability in initial ankle posture may still have influenced talar kinematics during the early landing phase. Fourth, electromyographic data were not collected simultaneously, precluding quantification of the relative contributions of passive ligamentous constraints and active neuromuscular control to anterior talar translation (Lin et al., 2022). Future studies integrating EMG and repeated valid landing trials with dual fluoroscopy are needed to assess neuromuscular contributions and within-subject kinematic variability.

## 5 Conclusion

This study demonstrates that static anterior ankle laxity is strongly associated with peak anterior talar translation during a single-leg landing task. Individuals with greater passive anterior laxity exhibited larger talar displacement under high-impact conditions, indicating that passive mechanical properties of the ankle are associated with dynamic bone motion. Quantified anterior drawer measurements provide an objective assessment of ankle laxity and may provide biomechanical information relevant to dynamic ankle stability. These findings provide further biomechanical evidence linking passive ankle laxity with *in vivo* talar motion during landing and highlight the need for future prospective studies to determine the clinical relevance of this association.

## Data Availability

The raw data supporting the conclusions of this article will be made available by the authors, without undue reservation.
